# Clinical and socio-demographic profile of children receiving pediatric palliative care in a tertiary hospital of a metropolitan city in India

**DOI:** 10.1007/s00431-024-05741-x

**Published:** 2024-09-13

**Authors:** Pratima Bisen, Poonam Wade, Pradnya Talawadekar, Sushma Malik, Mary Ann Muckaden, Surbhi Rathi, Jayita Deodhar

**Affiliations:** 1 Department of Pediatrics, Topiwala National Medical College and B.Y.L Nair Charitable Hospital, Mumbai, Maharashtra India; 2 Department of Palliative Medicine, Tata Memorial Hospital, Mumbai, India; 3 Homi Bhabha National Institute, Mumbai, India

**Keywords:** Palliative care, Complex chronic condition, Life-limiting conditions, Holistic care

## Abstract

Pediatric palliative care is a holistic care of children suffering from life-threatening or life-limiting illnesses and encompasses care of a child’s body, mind, and spirit and involves giving support to the family. According to the Global Atlas of Palliative Care, 6% of the global need for palliative care is in children. In order to provide palliative care, one needs to identify and diagnose the conditions requiring palliative care. There has always been a confusion in identifying pediatric conditions requiring palliative care. There is a lot of inconsistency in the diagnosis of such conditions particularly in pre-verbal patients. This study attempts to generate more data about the common palliative care conditions and complaints with which the children present to tertiary care hospitals. To study the socio-demographic details, clinical profile, CCC (complex chronic conditions) designation, and the ACT/ RCPCH (Association for Children with Life-threatening or Terminal Conditions and the Royal College of Pediatrics and Child Health) classification of children suffering from chronic conditions requiring palliative care. The study was conducted as a single-center retrospective observational study of pediatric patients enrolled for palliative care at a tertiary care hospital in a metropolitan city in India from 01.06.2021 to 31.06.2022. The total sample size was 400. The socio-demographic data and the clinical profile were recorded from the case records of all the 400 patients. Classification of the conditions was done as per the CCC as well as the ACT/ RCPCH classification system. The mean age in our study was 5.15 years and there was a slighter male (59.5%) preponderance. They presented in OPDs with acute symptoms such as fatigue and fever, and they had other symptoms like tightness of the body, constipation, seizures, and difficulty in swallowing. Majority of the children (55%) were suffering from neurologic and neuromuscular conditions as per CCC followed by hematologic and immunologic conditions (10%). Category 4 (irreversible but non-progressive conditions causing severe disability, leading to susceptibility to health) was reported as the most common category according to the ACT/RCPCH.

*   Conclusion:* Children suffering from chronic disease conditions requiring palliative care usually suffer from multiple symptoms which affect their daily life. As most of the patients belong to category 4 according to ACT/RCPCH which is an irreversible but non-progressive life-limiting condition, the course of the disease is prolonged, therefore requiring comprehensive care and services for a long time. It is necessary to establish more pediatric palliative care units to address the needs of such children.

**Table:** 

**What is known:** *• Pediatric palliative care is a specialized area within palliative care, which focusses on the needs of children with life-limiting illnesses.* *• Data on pediatric palliative care has largely been limited to oncological conditions. There is a paucity of literature documenting the needs among children suffering from non-cancerous chronic conditions.*	
**What is new:** *• This study provides vital information with respect to palliative care burden among children mainly suffering from non-oncological conditions.* *• It also provides clinical and socio-demographic profile of the children suffering from chronic life-limiting conditions requiring palliative care in a tertiary hospital setting in a LMIC (low- or middle-income country).*

**What is known:**

*• Pediatric palliative care is a specialized area within palliative care, which focusses on the needs of children with life-limiting illnesses.*

*• Data on pediatric palliative care has largely been limited to oncological conditions. There is a paucity of literature documenting the needs among children suffering from non-cancerous chronic conditions.*

**What is new:**

*• This study provides vital information with respect to palliative care burden among children mainly suffering from non-oncological conditions.*

*• It also provides clinical and socio-demographic profile of the children suffering from chronic life-limiting conditions requiring palliative care in a tertiary hospital setting in a LMIC (low- or middle-income country).*

## Introduction/background

Palliative care has been defined as the active holistic care of individuals across all ages with serious health-related suffering due to severe illness and especially of those near the end of life. It aims to improve the quality of life of patients, their families, and their caregivers [1]. Similarly, pediatric palliative care (PPC) is total care of a child’s body, mind, and spirit, and it also involves giving support to the family. PPC serves children suffering from various severe diseases and complex chronic conditions from birth to young adulthood while providing the necessary level of care to these patients which is also aligned to the goals of the patients and their families [2, 3].

Literature around the world indicates that the prevalence of children with a life-limiting condition was 66.4 per 10,000 in 2017/18 and is predicted to rise to 84.22 per 10,000 by 2030 [4]. The Global Atlas of Palliative Care at the End of Life [5] estimates that 6% of the global need for palliative care (based on mortality figures) is in children. In addition, the needs for palliative care have also been increasing among children suffering from cancers [6].

To provide palliative care, one needs to identify and diagnose the conditions requiring palliative care. Conventionally, a lot of literature can be found regarding pediatric palliative care in children with cancers [6, 7]. However, there is a paucity of literature on palliative care for non-oncology conditions. This is because there is still confusion regarding the identification of non-cancerous conditions requiring palliative care [7]. It is a very common practice to admit patients requiring palliative care in acute care hospitals. Hence, it is very important to understand and be aware of the main conditions that lead to the hospitalization of such patients. The identification of these conditions in children is further complicated by the fact that they/ caretakers are unable to give a complete and reliable history about their problems. Pre-verbal patients do not even communicate their complaints. This leads to inconsistency in the diagnosis of various conditions as well as difficulty in the identification of common complaints such as pain [8].

The purpose of our study is to examine the common conditions of children presenting to a pediatric palliative care clinic in a general tertiary care hospital in a LMIC (low- or middle-income country) setting like India. We are contributing to the available literature — which may be less in low-resource settings. The objectives were to assess the socio-demographic details of children suffering from chronic conditions requiring palliative care, to determine the clinical profile and complex chronic conditions (CCC) according to the CCC system designation of children with chronic conditions requiring palliative care, and to examine the classification of the disease conditions according to the Association for Children with Life-threatening or Terminal Conditions and the Royal College of Pediatrics and Child Health.

## Materials and methods

### Study design, setting, and study population

We conducted a single-center retrospective observational study of all pediatric patients (aged from birth till 12 years of age) enrolled for palliative care in the pediatric palliative care clinic. The referrals to the clinic were from pediatric ward, pediatric OPD, pediatric surgery ward, hematology clinic, pediatric intensive care unit (PICU), and neonatal intensive care unit (NICU) at a tertiary care hospital in a metropolitan city in India from 01.06.2021 to 31.06.2022. The study was conducted after seeking approval from the Ethics Committee of the Institute (Ref. No: ECARP/2022/151).

### Eligibility criteria

All patients referred to PPC service from the above-mentioned settings in the study period.

### Data collection

The data was collected from prospectively maintained medical records in the PPC clinic. Variables noted were (a) socio-demographic variable, (b) symptoms, and (c) clinical — according to the systems, i.e., complex chronic conditions (CCC) classification system as well as the Association for Children with Life-threatening or Terminal Conditions and the Royal College of Pediatrics and Child Health.

### Data analysis

The data collected was entered in the Microsoft Excel worksheets. Descriptive statistics were used to analyze the socio-demographic and clinical data. Relevant tables, pie charts, and bar diagrams were also used to represent the results.

#### Complex chronic conditions (CCC) classification system

Life-limiting illnesses are classified in nine CCC categories: neuromuscular (brain/spinal cord malformation, intellectual disability, CNS disease, cerebral palsy, epilepsy, muscular dystrophy), cardiovascular (heart malformations, cardiomyopathies, and dysrhythmias), cancer, congenital anomalies (chromosomal abnormalities, bone/joint abnormalities, diaphragm/abdominal abnormalities, other abnormalities), respiratory (respiratory malformations, chronic respiratory disease, cystic fibrosis), gastrointestinal (congenital anomalies, liver disease, inflammatory bowel disease), metabolic (amino acid, carbohydrate, lipid, storage disorders, other disorders), hematologic, (sickle cell disease, anemias, hereditary immunodeficiency, HIV), and renal (congenital abnormalities, chronic renal failure) [9].

#### Association for children with life-threatening or terminal conditions and the royal college of pediatrics and child health categorization [10]

Life-limiting and life-threatening conditions affecting children can be divided broadly into four groups:

1. Conditions where curative treatment may be feasible but can fail, e.g., cancer.
2. Conditions requiring intensive long-term treatment aimed at maintaining the quality of life allowing participation in normal activities, e.g., severe immunodeficiency, cystic fibrosis, muscular dystrophy.
3. Progressive conditions without curative treatment options where treatment is exclusively palliative and may commonly extend over many years, e.g., progressive metabolic disorders.
4. Irreversible but non-progressive conditions causing severe disability, e.g., cerebral palsy, where there remains a high risk of an unpredictable life-threatening event.

## Results

A total of 400 children were referred to the PPC clinic in the study duration. The mean age of the patients receiving PPC was 5.15 years. Of the 400 study subjects, 238 (59.5%) were males (Table 1). More than half of the children (231, 57.8%) belonged to Hindu families followed by 160 (40%) children who belonged to Muslim families (Table 1). Almost half of the patients (186, 46.5%) had a normal nutritional status, while 63 (15.8%) had severe acute malnutrition. About 15 (3.8%) and 9 (2.3%) children were overweight and obese respectively.

**Table 1:** Patients characteristics and socio-demographic profile

Characteristics	Frequency (*n*)	Percentage (%)
Entire	400	100
Age		
< 1 y	59	14.8
1–< 5 y	132	33
5–< 10 y	138	34.5
10–≤ 12	71	17.8
Gender		
Male	238	59.5
Female	162	40.5
Religion		
Hindu	231	57.8
Muslim	160	40.0
Buddhist	6	1.5
Christian	3	0.8
Primary caregiver		
Mother	397	99.3
Aunt	2	0.5
Grandmother	1	0.3
Socio-economic classification as per Kuppuswamy scale		
Upper	2	0.5
Upper middle	25	6.3
Lower middle	40	10
Upper lower	320	80
Lower	13	3.3

Majority of the participants (320, 80%) belonged to the upper lower socio-economic class (Modified Kuppuswamy scale) [11] followed by 65 (16.3%) participants who belonged to the middle class. It was also found that mothers were the primary caregivers for almost all (397, 99.3%) of the study participants (Table 1).

The participants in the study were found to suffer from multiple complaints at the same time. As evident from Table 2, the most prevalent complaint reported by the children was lack of energy/ fatigue (283, 70.8%) followed by fever (188, 47%) and constipation (156, 39%).

**Table 2:** Symptomatology of patients

Symptom	Frequency (*n*)	Percentage (%)
Lack of energy (fatigue)	283	70.8
Fever	188	47
Constipation	156	39
Tightness of body/ spasticity	156	39
Seizures	138	34.5
Difficulty in swallowing	131	32.8
Sleep difficulty	130	32.5
Eating appetite	119	29.8
Cough	117	29.3
Irritable	114	28.5
Shortness of breath	111	27.8
Drowsy	105	26.3
Looseness of body/ hypotonia	73	18.3
Pain	69	17.3
Vomiting	64	16
Changes in skin (rash and discoloration)	45	11.3
Bleeding	35	8.8
Painful urination	34	8.5
Mouth sore	30	7.5
Dry mouth	27	6.8
Excessive sweating	20	5
Diarrhea	18	4.5
Itching	9	2.3

**Fig. 1:**
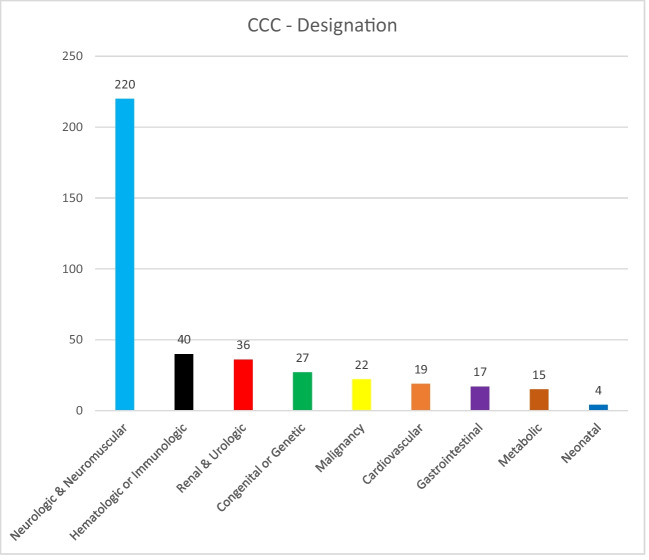
Categorization of patient according to CCC (complex clinical conditions) classification

More than half (220, 55%) of the patients belong to the neurologic and neuromuscular condition category as per the CCC categorization (Fig. 1). Only 40 (10%) patients belonged to the hematologic and immunologic conditions followed closely by the renal and urologic conditions (36, 9%). The least number of patients belonged to the neonatal condition category (4, 1%).

**Fig. 2:**
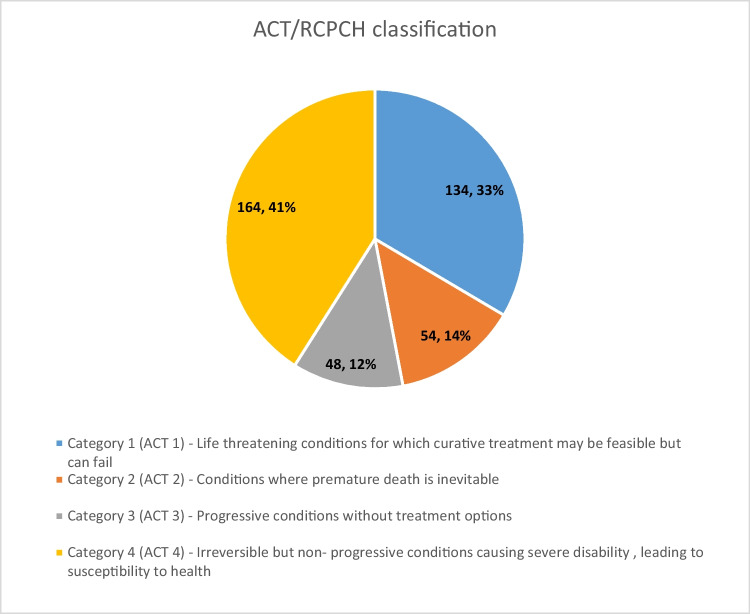
Categorization of patient according to ACT/RCPCH (Association for Children with Life-threatening or Terminal Conditions and their Families and the Royal College of Pediatrics and Child Health) classification

When classified according to the ACT/RCPCH classification, 164 (41%) patients were assigned to ACT-4, 134 (33%) to ACT-1, 54 (13.5%) to ACT-2, and 48 (12%) to ACT-3 (Fig. 2).

## Discussion

In our study (Table 1), the mean age was 5.15 years, while a multi-center study conducted by Muckaden et al. in Western India reported the average age of children as 9.3 years [12]. A study by Harputluoğlu et al. in Turkey found the mean age to be around 5.2 years [13]. Another study in Turkey by Ayar et al. also reported a similar finding with median age as 4.42 years [14]. In our study, there were 59.5% males (Table 1) which was similar to the studies conducted by Hayden et al. [15] and Muckaden et al. [12] who reported 51% and 60.6% subjects as males respectively. Harputluoğlu et al. and Ayar et al. also described similar findings with 53.6% and 50.3% of subjects being males [13].

In our study, about 53.7% of patients were malnourished with 47.6% being undernourished, 3.8% being overweight, and 2.3% being obese. These findings were similar to the study conducted by Radhakrishnan et al. who found that 153 out of 295 (52%) patients were well-nourished, 130/295 (44%) patients were undernourished, and 12/295 (4%) patients were obese [16]. With respect to the prevalence of obesity, our study findings presented a distinct contrast as compared to another study from the USA conducted by Orgel et al. which reported almost 14% obesity [17]. This difference could be due to the influence of socio-economic status and cultural habits on the nutritional status.

In our study, majority of the patients (83.3%) belonged to lower class (Modified Kuppuswamy classification). A different trend was observed in a study conducted by Kumar et al. where more than half of the participants belonged to the middle class [18]. This difference could be due to the fact that majority of the participants in the current study were migrants from different cities and states.

Almost all the caregivers (99.3%) in the current study were mothers which is very similar to the study conducted by Pegah Piran et al. [19] in Iran where most of the caregivers were the mothers. This can be explained by the traditional and cultural expectations from women to be the primary caregivers of their children.

In our study, the most prevalent symptoms were fatigue or lack of energy, fever, tightness of the body (spasticity), constipation, seizures, and difficulty in swallowing (Table 2). The study by Ayar et al. also reported fever in many patients, and almost 42.7% admissions were due to infections [14]. In another study done by Feudtner et al., the most prevalent symptoms were pain, lack of energy, irritability, drowsiness, and shortness of breath [20]. Pain was reported by a relatively lesser number of patients in our study (17.3%). One of the reasons for this could be the fact that most of these children were suffering from chronic conditions such as neurological diseases. Most of the hospital admissions in our study were due to the acute symptoms that the study subjects were suffering from. HarnEnz et al. also observed a similar trend in their study where they mentioned that over half of the admissions were for acute relief of symptoms [21]. Research from around the world shows that children suffering from life-threatening illnesses often report many symptoms such as fatigue, irritability, shortness of breath, and pain [22–25]. Some authors have also reported that children with progressive conditions usually presented with behavioral symptoms such as agitation [26], feeding problems [27], and sleep difficulties. We also found spasticity, seizures, and constipation as common symptoms which was different from other studies which could be because majority of our patients were suffering from neurological disorders, while the participants in these studies were cancer patients [22–25].

As per the current study, the most prevalent complex clinical conditions were neurological [220 (55%)] followed by hematological [40 (10%)], renal [36 (9%)], congenital causes [27( 6.8%)], and malignancy [22 (5.5%)] which was nearly similar to with study conducted by Feudtner et al. which demonstrated cardiovascular, neurological, and malignancy as the most common CCC group [20]. The study by Harputluoğlu et al. also found cerebral palsy as the most common diagnosis (39.2%) among their study subjects [13]. Another study by Feudtner et al. also reported the prevalent complex chronic conditions as gastrointestinal (357 [71.3%]), neurologic (289 [57.7%]), and cardiovascular (310 [61.9%]) conditions [28]. Ayar et. al., on the other hand, noted the most common diagnosis as genetic and congenital syndromes (20.7%) [14]. These differences could be because of the specialized pediatric OPDs that are functional in different hospitals.

The majority (164, 41%) of the patients in the study belonged to the “irreversible but non-progressive conditions causing severe disability” (ACT-4) according to the ACT/ RCPCH. Almost 134 (33%) participants belonged to the ACT-1 category (life-threatening conditions for which curative treatment may be feasible but can fail). As per a study by Bender et al., 36% of patients were assigned to ACT-3, 34% to ACT-4, 26% to ACT-1, and 4% to ACT-2 [29].

The limitation of the study was that we could not adequately compare our findings with other studies based on chronic conditions (other than malignancy) as literature regarding the same is limited, particularly in the Indian context. We had to rely mainly on cancer-related studies. As the pediatric department usually has different divisions such as pediatric wards, outpatient departments, ICUs, special clinics (neurology, respiratory, nephrology), and neonatal units, it sometimes poses difficulty for the pediatric palliative care unit to cover all the patients across different sections. Sensitization of different healthcare workers with regard to pediatric palliative care can raise awareness which will pave the way for efficient management of palliative care patients.

## Conclusion

This study provides essential insights with respect to the socio-demographic profile and symptoms of children suffering from life-limiting illnesses requiring palliative care. Patients suffering from chronic disease conditions requiring palliative care suffer from multiple symptoms which affect their daily life, and knowledge of these symptoms is essential for their management. Majority of patients were male with a median age of 5.15 years. Most of them were from a poor financial background and are malnourished and the mothers or women of the house usually carry the entire caregiving burden. As most of our patients belong to the neurological and neuromuscular CCC category and ACT-4 according to ACT/RCPCH, the course of the disease is prolonged, and so they require holistic care and services for a long time. The establishment of specialized palliative care units integrated with pediatric departments in the hospitals can be very effective in the implementation of different palliative care components for all the children who need such services. The current study identified the various conditions that warrant palliative care. It is of utmost importance for similar units to be set up to address the palliative care needs for these children.

## Data Availability

No datasets were generated or analysed during the current study.
